# Elevation of C-reactive protein, P-selectin and Resistin as potential inflammatory biomarkers of urogenital Schistosomiasis exposure in preschool children

**DOI:** 10.1186/s12879-019-4690-z

**Published:** 2019-12-19

**Authors:** Theresa N. Chimponda, Caroline Mushayi, Derick N. M. Osakunor, Arthur Vengesai, Eyoh Enwono, Seth Amanfo, Janice Murray, Cremance Tshuma, Francisca Mutapi, Takafira Mduluza

**Affiliations:** 1 University of Zimbabwe, Biochemistry Department, P. O. Box MP 167, Mt Pleasant, Harare, Zimbabwe; 2 Mashonaland Central Provincial Health Office, Ministry of Health & Child Care, Harare, Zimbabwe; 3 Statistics Department, University of Zimbabwe, P. O. Box MP 167, Mt Pleasant, Harare, Zimbabwe; 4 Centre for Immunity, Infection and Evolution, Institute of Immunology and Infection Research, University of Edinburgh, Ashworth Laboratories, King’s Building Charlotte Auerbach Road, Edinburgh, EH9 3FL UK; 5 Centre for Immunity, Infection and Evolution, Usher Institute of Population Health Sciences and Informatics, University of Edinburgh, Edinburgh, UK; 6 NIHR Global Health Research Unit Tackling Infections to Benefit Africa at the University of Edinburgh, Edinburgh, UK

**Keywords:** C-reactive protein, Resistin, P-selectin, Inflammatory biomarkers, Schistosomiasis

## Abstract

**Background:**

Schistosomiasis is known to induce inflammatory immune responses. C-reactive protein (CRP), resistin and P-selectin are serological inflammatory markers that rise during the acute stages of infection. Here, we propose such inflammatory biomarkers have a potential for use in urogenital schistosomiasis diagnostic screening for exposure and infection in preschool-aged children.

**Methods:**

As part of a larger study on urogenital schistosomiasis, 299 preschool children aged 1–5 years were included in this cross-sectional study. Parasitological diagnosis was conducted using urine filtration for *Schistosoma haemtobium* infection, and Kato Katz for *S. mansoni* infection. Serum levels of P-selectin, resistin, CRP, and antibodies against *S. haematobium* cercarial antigen preparation (CAP) and soluble worm antigen preparation (SWAP) were measured by ELISA.

**Results:**

Of the 299 participants, 14% were egg positive for *S. haematobium*. Serology showed 46 and 9% of the participants to have been exposed to *S. haematobium* cercarial antigens and adult worm antigens, respectively. Levels of P-selectin were significantly higher in participants infected with *S. haematobium* (egg-positive) than in uninfected participants (*p* = 0.001). Levels of P-selectin were also higher in those exposed to cercarial antigen than in unexposed participants (*p* = 0.019). There was a positive correlation between P-selectin and infection intensity (*r* = 0.172; *p* = 0.002), as well as with IgM responses to CAP and SWAP (*r* = 0.183; *p* = 0.001); (*r* = 0.333; *p* < 0.0001) respectively. CRP significantly correlated with IgM responses to CAP (*r* = 0.133; *p* = 0.029) while resistin correlated with IgM responses to CAP and SWAP (*r* = 0.127; *p* = 0.016); (*r* = 0.197; *p* = 0.0004). CRP levels were higher in those exposed to cercarial and adult worm antigens than unexposed participants (*p* = 0.035); (*p* = 0.002) respectively, while resistin was higher in participants exposed to cercarial antigen than unexposed participants (*p* = 0.024).

**Conclusion:**

In this preschool population, P-selectin is significantly associated with urogenital schistosome infection and intensity; hence a potential biomarker for infection diagnosis and disease monitoring. The inflammatory biomarkers (P-selectin, Resistin and CRP) were significantly higher in participants exposed to cercarial antigens than unexposed individuals indicating an underlying inflammatory environment.

## Background

Schistosomiasis is a parasitic disease that is second to malaria in terms of public health impact in sub-Saharan Africa, and affects in excess of 200 million individuals in more than 70 countries [8]. The disease is caused by digenetic trematodes of the genus *Schistosoma*. *S. haematobium* is responsible for the urogenital form of the disease and affects over 112 million individuals, resulting in 150,000 deaths annually in sub-Saharan Africa [10]. Symptoms of the disease include haematuria, anaemia [17], fibrosis of the bladder and ureter, as well as kidney damage during the later stages of infection [22]. Diagnosis of schistosomiasis include parasitology for the detection of eggs in urine or feaces, antibody and cytokine detection in serum or plasma, DNA and RNA detection as well as use of biomarkers such as chitinase-3-like-1 protein [3], antigens (circulating anodic and cathodic antigens (CAA and CCA) [6] and lipopolysaccharide-binding protein [24].

Inflammatory markers can also serve as biomarkers of infection especially for asymptomatic disease cases. Moreover the biomarkers can possibly predict disease outcome [16] and can be useful for the detection of possible infection in population groups that rarely acquire heavy infection; especially preschool-aged children during the early years when they first get exposed and infected. Inflammatory markers such as C-Reactive Protein (CRP), fibrinogen, may be used to detect acute inflammation which can be indicative of a particular disease or can be used as a marker of response to treatment [32]. Measuring inflammatory markers in schistosomiasis is particularly important, as there is a persistent acute phase response, which is caused by chronic infection [5].

CRP is persistently produced during infection in the acute phase response via tumor necrosis factor α (TNF- α), interleukin -1β (IL 1 β) and IL-6 [5]. P-selectins have a critical role in the progression of chronic liver disease caused by schistosome parasites as compared to the other selectins [14]. Resistin has damaging effects in multiple helminth infections by mediating pathogenic inflammation and impeding parasite clearance [13]. Early diagnosis of preschool-aged children is particularly important as infection in these children can become cumulative, increasing with age [34]. Here, we focus on CRP, resistin and P-selectin which herein refer to as inflammatory markers. The aim of this study was to determine the levels of these inflammatory biomarkers in preschool-aged children living in high schistosome transmission areas. We hypothesised that schistosome-infected preschool-aged children have higher levels of inflammatory biomarkers compared to uninfected participants. Also that schistosome exposure or infection intensity in preschool-aged children is associated with high levels of inflammatory biomarkers. Our analysis set to evaluate whether 1) levels of inflammatory biomarker are higher in schistosome infected children than uninfected children, 2) schistosome infection intensity is associated with CRP, resistin and P-selectin, 3) Immunoglobulin M (IgM) response to CAP or SWAP is associated with levels of CRP, resistin and P-selectin.

## Methods

### Study area and population

The study was conducted in 299 preschool children (≤5 years of age) as part of a larger longitudinal study investigating the immunological responses of paediatrics at first infection with schistosomiasis, in the Shamva district of the Mashonaland Central Province of Zimbabwe. The district has been reported to have a high prevalence of *S. haematobium* infection (≥50%) [19]. The villagers are mainly subsistence farmers and carry out water-related activities such as farming and gardening. Mothers usually go with their children as they carry out these activities.

### *S. haematobium* and *S. mansoni* diagnosis

Urine sample was collected from each participant on three consecutive days and a stool specimen collected on a single day from each participant. *S. haematobium* infection was determined using urine filtration technique [20]. In order to exclude *S. mansoni infection,* Kato Katz [15] was used to detect eggs in faeces. Egg-positive individuals for *S. haematobium* and *S. mansoni* had at least an egg present in urine and stool, respectively. Egg-positive participants were treated with a single dose of praziquantel at the standard 40 mg/kg body weight [33].

### Sera collection

About 5 ml of venous blood was collected from each participant into plain tubes (BD vacutainer, Fisher Scientific) and allowed to sit for 1 h at room temperature (25 °C) to allow the blood to clot. The clot was removed by centrifugation at 2555 g for 10 min and the serum aliquoted into 1.5 ml tubes and stored at − 20 °C until assayed.

### Antibody assays

In this study, only IgM levels were measured. In a schistosome exposure study (where anti-cercarial or anti-egg immune responses were measured) in young children (up to 6 years old), it was shown that IgM is the predominant isotype [34]. Furthermore, IgM antibodies are produced early in infection [31]. The indirect enzyme linked immunosorbent assay (ELISA) was done as in [21]. Cercarial antigen preparation (CAP) and soluble worm antigen preparation (SWAP) were obtained from the Theodor Bilharz Institute (Giza, Egypt). Initial titrations with different antigen concentrations and secondary antibody dilutions were used to determine optimal conditions for the final ELISA. 96-well high binding plates (Greiner bio-one, Germany) were coated with 100 μl/well of 1 μg/ml antigen (CAP or SWAP) in 60 mM carbonate buffer (pH 9.6). The plates were sealed with parafilm and incubated overnight at 4 °C. The contents of the plates were discarded and washed 3 times with wash buffer (Phosphate buffered saline (PBS, 0.1% Tween 20). The plates were blotted on multiwipe paper and then blocked with 200 μl of blocking buffer (PBS, 5% skimmed milk, 0.03% Tween 20) for 2 h at room temperature. The plates were washed and blotted as stated before. Sera from the participants as well as negative control samples (pooled sera from donors who had never travelled to an *S. haematobium* endemic area) were diluted at 1:100 in blocking buffer. A 100 μl of diluted sera was added to the wells in duplicate and the plates were incubated at room temperature for 2 h. The washing procedures were repeated as before and 100 μl of the conjugated secondary antibody (IgM) (horseradish peroxidise (HRP) conjugated) (Sigma, Germany), diluted at 1:1000 in blocking buffer, was added and the plates were incubated at room temperature for 2 h. The plates were washed, followed by the addition of substrate (o-phenylenediamine dihydrochloride (OPD), and were incubated for 20 min at room temperature, in the dark. Absorbance was measured using a microplate reader (Biotek, USA) at 450 nm. Samples were assayed in duplicate, and a blank control was included (in duplicate) on each plate and the background absorbance was subtracted from all readings.

### Measurement of inflammatory biomarkers

Serum levels of CRP, resistin and P-selectin were determined by the sandwich ELISA method (Duoset ELISA, DY1707, DY 1359 and DY 137 respectively) according to the manufacturer’s (R&D Systems) instructions. The ELISA was optimised in order to determine the best conditions for the final assay. Briefly, 100 μl each of serum sample (× 200 dilution factor) was diluted in 13 mM phosphate buffered saline, (pH 7.2) containing 1% bovine serum albumin. The samples and standards were added in duplicate to 96-well high binding plates (Greiner bio-one, Germany) that had been coated overnight with 2 μg/ml of capture antibody (mouse anti-human CRP, resistin or P-selectin capture antibody) and incubated for 2 h at room temperature. The plates were washed four times with 0.05% Tween 20 in PBS (pH 7.2), 100 μl of detection antibody (90 ng/ml Biotinylated mouse anti human CRP, 250 ng/ml resistin detection antibody or 20 ng/ml P-selectin detection antibody) was added and incubated for 2 h at room temperature (25 °C). The washing was repeated as before, streptavidin HRP was added, and the plate incubated for 20 min at room temperature in the dark. The plates were washed once again and 100 μl of the substrate solution OPD was added and incubated for 20 min at room temperature. The absorbance was measured using a microplate reader (Biotek, USA) at 450 nm.

### Ethical considerations

Blood, urine and stool specimens were obtained from willing children who agreed to participate in the study, following the signing of informed consent forms by their parents and guardians. Ethical approval for the study was obtained from the Medical Research Council of Zimbabwe Approval; MRCZ/A/1964. In addition, Provincial Medical Director, councillors and village headmen granted permission for the study.

### Statistical analysis

Statistical analyses were conducted using STATA version 13 and GraphPad Prism version 5.0 (GraphPad Software, INC). The cut-off for determining those participants who were exposed to cercarial or adult worm antigens was determined by calculating the mean of the negative control samples plus two standard deviations [34]. Samples with absorbance values (optical density (OD)) greater than the cut-off value were considered sero-positive. Differences in IgM response levels to CAP or SWAP between schistosome-positive or negative individuals as determined by parasitology were analysed using the Mann-Whitney U test. Spearman’s rank method was used to assess the relationships between inflammatory biomarkers with schistosome infection intensity or IgM response to CAP or SWAP. The Mann-Whitney U test was used to compare the inflammatory biomarker levels according to infection status or exposure to cercarial or adult worm antigen status. For all analysis a *p* values < 0.05 was considered significant.

## Results

### Parasitology and antibody response levels

**Fig. 1:**
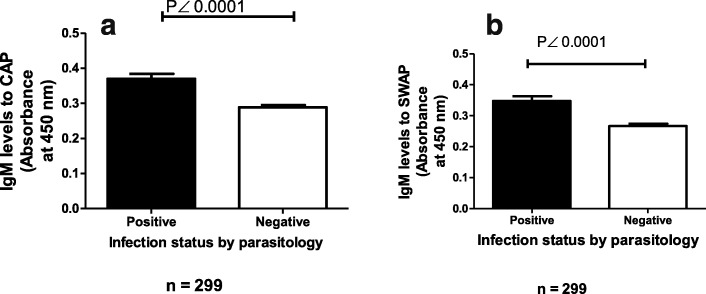
IgM response levels to Cercarial Antigen Preparation (CAP) (**a**) and Soluble Worm Antigen Preparation (SWAP) (**b**) according to infection status as determined by parasitology. Schistosome specific IgM antibody levels to CAP and SWAP were determined by Enzyme Linked Immunosorbent Assay (ELISA) and the experiment was done in duplicate. The black bar represents responses to CAP or SWAP for those positive for *S. haematobium* while the open bar represents those negative. Mean IgM responses are shown with error bars representing standard error of the mean. The Mann Whitney U-test was used to test the difference in IgM response levels to CAP or SWAP between egg positive and egg negative individuals with *p* values less than 0.05 being significant

Of the 299 participants, 53% (157) were male and 47% (142) were female **(**Table 1**)**. 14% of the participants (42) were egg positive for *S. haematobium* infection. The overall mean of *S. haematobium* infection intensity was 2.81 eggs/10 mL urine (SD =19.19). The overall mean of IgM antibody response to CAP and SWAP were 0.23 OD (SD = 0.033) and 0.22 OD (SD = 0.095), respectively. Based on the cut-off for CAP (0.29) and SWAP (0.41), the proportion of seropositive participants was higher for those exposed to cercarial antigens, 139 (47%) than those exposed to adult worm antigens, 26 (9%). IgM responses to CAP or SWAP were plotted according to infection status as determined by parasitology (Fig. 1). In both cases level of IgM responses to CAP or SWAP were significantly higher for those positive for *S. haematobium* than negative individuals (*p* < 0.0001). The relationship between IgM responses to CAP or SWAP with infection intensity was analysed by Spearman’s rank method. There was a positive association between IgM responses to CAP or SWAP and infection intensity (*r* = 0.287; *p* < 0.0001); (*r* = 0.298; *p* < 0.0001) respectively.

**Table 1:** Demographic summary of the study population

*S. haematobium* infection status	Male (n, %)	Female (n, %)	Total (299)
Negative	135(45.2)	122(40.8)	257(86)
Positive	22(7.3)	20(6.7)	42(14)
Total	157(53)	142(47)	299(100)

Data is presented as n (%)

### Levels of inflammatory markers according to infection or exposure status

**Fig. 2:**
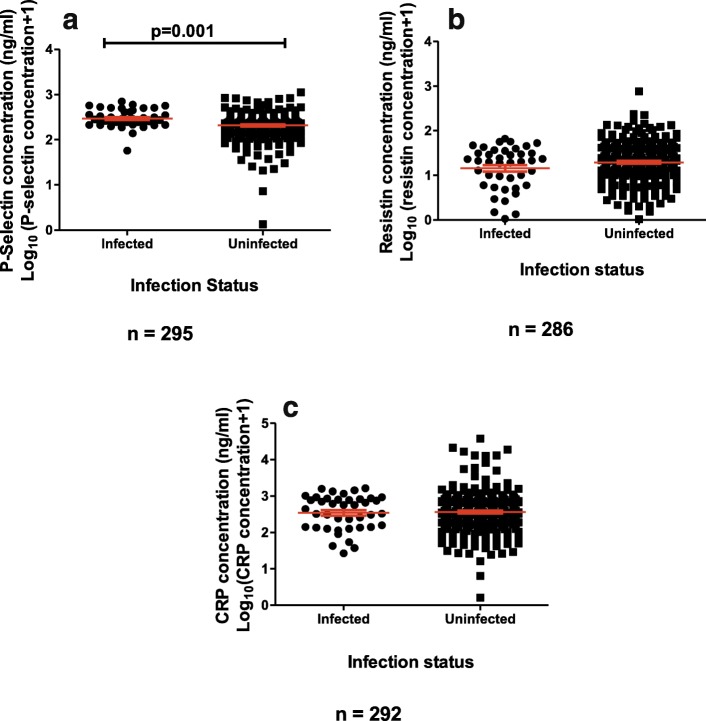
Serum levels of inflammatory marker according to infection status. The levels of P-selectin (**a**), Resistin (**b**), and C-Reactive Protein (CRP) (**c**) were determined using serology and were plotted aginst infection status. The assay was carried out in duplicate. Differences in levels of inflammatory marker for the uninfected and infected were analysed using the Mann Whitney U-test with *p* values less than 0.05 being significant. The orange bar represents the median value

**Fig. 3:**
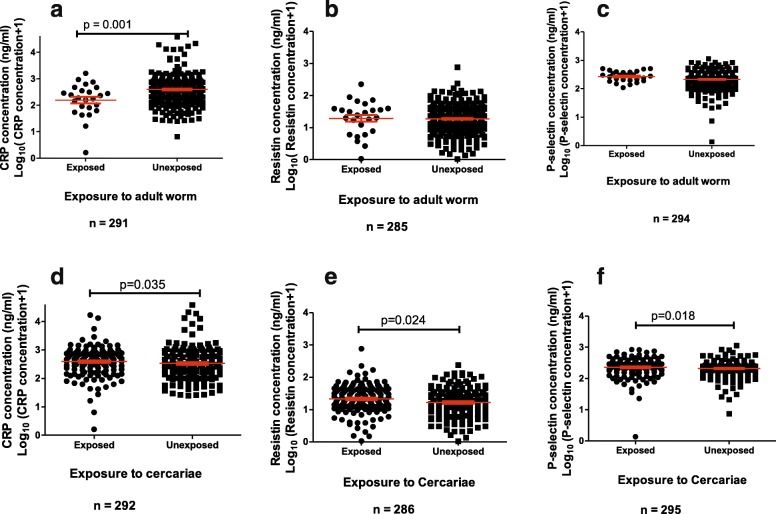
Serum levels of inflammatory marker according to exposure status. The levels of P-Selectin, C-Reactive Protein (CRP) and Resistin were analysed by serology with the assay carried out in duplicate. Differences in levels of inflammatory marker for participants exposed to Soluble Worm Antigen Preparation (SWAP) (**a**, **b**, **c**) or Cercarial Antigen Preparation (CAP) (**d**, **e**, **f**) or and those unexposed were analysed using the Mann Whitney U-test. The orange bar represents the median value

The levels of Resistin, P-selectin and CRP for the schistosome-infected and uninfected were detected using ELISA and plotted in (Fig. 2). Only P-selectin levels were higher in infected participants than the uninfected participants (*p* = 0.001). Levels of CRP were significantly higher for those exposed to adult worm (A) or cercarial (D) antigens (*p* = 0.035); (*p* = 0.002) respectively (Fig. 3). Levels of Resistin (E) and P-selectin (F) were higher in those exposed to cercarial antigens (*p* = 0.024); (*p* = 0.019), respectively.

### The relationship of CRP, Resistin and P-selectin with infection intensity or IgM responses to CAP or SWAP

Associations between inflammatory marker with infection intensity or IgM responses to CAP or SWAP were analysed using Spearman’s rank test and the results are summarised in (Table 2). There was a significant positive association between P-selectin and infection intensity (*r* = 0.172; *p* = 0.002) while resistin and CRP did not have a significant association with infection intensity. A significant positive association was observed between all the inflammatory markers and IgM responses to CAP, CRP (*r* = 0.133; *p* = 0.029); P-selectin (*r* = 0.183; *p* = 0.001); Resistin (*r* = 0.127; *p* = 0.016). P-selectin and Resistin had significant positive association with IgM responses to SWAP (*r* = 0.333; *p* < 0.0001) (*r* = 0.197; *p* = 0.0004) respectively while CRP did not have an association with IgM response to SWAP.

**Table 2:** Correlation between inflammatory marker with Infection intensity or IgM response to CAP or SWAP

Inflammatory marker	Infection Intensity r (*p* value)	IgM response to CAP r (*p* value)	IgM response to SWAP r (*p* value)
CRP	0.009 (0.437)	**0.133 (0.029)**	−0.054 (0.179)
Resistin	−0.073 (0.111)	**0.127 (0.016)**	**0.197 (0.0004)**
P-Selectin	**0.172 (0.002)**	**0.183 (0.001)**	**0.333 (< 0.0001)**

r is the spearman’s correlation coefficient. Significant results for *p* < 0.05 are in bold

## Discussion

Studies have linked inflammatory biomarkers to various diseases; resistin has been linked with diabetes [13], CRP with malaria [12] and P-selectin with systemic inflammatory response syndrome [23]. However, there is paucity of data on their role in schistosomiasis. Wami, [30] evaluated the role of resistin and CRP in schistosomiasis in children 1–10 years old and CRP was observed to be one of the inflammatory biomarkers in children with schistosomiasis. Recommendations were made for further studies on a larger cohort of preschool-aged children. Our current study sought to evaluate the potential of CRP, resistin and P-selectin as a diagnostic tool for urogenital schistosomiasis in preschool-age children. Children in this age group are usually excluded from schistosome-control programs due to lack of understanding of infection and disease dynamics, thus, there is a need for sensitive indirect diagnostic markers. Identification of additional biomarkers for detection of schistosomiasis related morbidity would be an invaluable tool for schistosome control programs. Especially in schistosome infections where such markers may facilitate timely treatment to prevent severe morbidity [28].

P-selectin levels were significantly higher in the schistosome infected than in the uninfected participants. The levels of P-selectin were also significantly higher for participants exposed to cercarial antigens. A significant positive association was found between P-selectin and IgM responses to SWAP and CAP. To the best of our knowledge, this is the first study investigating the relationship between *S. haematobium* infection and P-selectin in preschool-aged children. As a neglected disease in this age group, whose immune responses are not yet fully developed and matured [9], early identification of inflammatory biomarkers could indicate exposure to schistosomiasis. P-selectins are molecules involved in chronic and acute inflammation processes. P-selectin is considered a biomarker of endothelial dysfunction [11]. Schistosomiasis (infection and egg deposition) causes endothelial damage [18] and this may result in increased levels of p-selectin. P-selectin also has a role in leucocyte recruitment and is associated with pro-inflammatory cytokines [27] hence,it is likely increased in schistosome-infected participants. The mechanism involves P-selectin interaction with a ligand known as P-selectin glycoprotein ligand-1 (PSGL-1) which induces the activation of leucocytes as well as cytokine production by the leucocytes. Cytokines such as TNF-α, interleukin (IL)-1β, IL-6, IL-8, IL-12 are increased by P-selectin mediated cell adhesion [26]. Nitric oxide (NO) production has been shown to downregulate P-selectin expression [1].

CRP levels were significantly higher in participants exposed to cercarial or adult worms than unexposed individuals but not when compared between those infected and uninfected participants as determined by parasitology. Our results may be explained by the fact that in most cases of antibody-positivity, egg-negativity reveals the failure of insensitive microscopy to detect eggs in subjects who are lightly-infected [25]. The inflammatory markers may have been raised in these individuals who were missed by parasitology. Previous schistosome studies on CRP focused on children > 5 years [4, 5] have shown a positive correlation between CRP and schistosome infection.

Focusing on several biomarkers instead of a single indicator may aid in the identification of the most effective biomarkers of inflammation during schistosomiasis [29]. Resistin was also investigated and a positive association with IgM responses to CAP or SWAP was observed. Participants exposed to cercarial antigens also had higher levels of resistin than unexposed participants. However, there were no significant differences in levels of resistin between infected and uninfected participants. Also a negative association was found between infection intensity and resistin levels. This is in contrast to several studies have reported elevated resistin levels in various inflammation-related diseases like rheumatoid arthritis, inflammatory bowel disease [7] and failed renal allografts [2]. Patients infected with soil-transmitted helminths were also reported to have elevated serum Resistin, which positively correlated with parasite burden and pro-inflammatory cytokines [13].

Our results have shown an underlying inflammatory environment for preschool-aged children exposed to schistosome antigens. Longitudinal studies to determine changes in the inflammatory markers with infection and treatment would improve the study.

## Conclusion

The study has demonstrated that P-selectin is a potential biomarker of diagnosis for urogenital schistosomiasis in preschool-aged children where infection is low, and during the period they are exposed to schistosome. Further research in preschool-aged group in other geographical areas is recommended. This will enable the validation of our result and the potential use of P-selectin as a diagnostic marker. More investigations characterising the impact of schistosomiasis exposure or infection on the inflammatory pathways should be explored, especially in the age group getting infection during the early develpmental age.

## Data Availability

The datasets are available from the corresponding author on reasonable request.

## Abbreviations

CAA: Circulating anodic antigen
CAP: Cercarial antigen preparation
CCA: Circulating cathodic antigen
CRP: C-Reactive protein
ELISA: Enzyme linked immunosorbent assay
HRP: Horse radish peroxidise
IgM: Immunoglobulin M
IL: Interleukin
NO: Nitric oxide
OD: Optical density
OPD: O-phenylenediamine dihydrochloride
PBS: Phosphate buffered saline
PSGL-1: P-selectin glycoprotein ligand-1
SWAP: Soluble worm antigen preparation
TNF- α: Tumor necrosis factor α
